# Identification of plasminogen activator urokinase receptor-related non-coding RNA and immune prognostic signature for non-small cell lung cancer

**DOI:** 10.1016/j.clinsp.2025.100711

**Published:** 2025-06-24

**Authors:** Yuan Cheng, Shihao Bao, Hao Zhang, Yifan Zhang, Xianjie Li, Hanyi Li, Xuewang Jia, Qianqian Sun, Zuoqing Song

**Affiliations:** a Department of Thoracic Surgery, North China University of Science and Technology Affiliated Hospital, Tangshan, Hebei, PR China; b Department of Lung Cancer Surgery, Tianjin Medical University General Hospital, Tianjin, PR China; c Department of intensive care unit, The Second Hospital of Tangshan, Tangshan, Hebei, PR China

**Keywords:** Non-small cell lung cancer, Plasminogen activator urokinase receptor, Non-coding RNAs

## Abstract

**Objective:**

To explore the relevant regulation of Plasminogen Activator Urokinase Receptor (PLAUR) by non-coding RNAs (ncRNAs) and the relationship between PLAUR and immune cell infiltration and biomarkers.

**Methods:**

This is a cross-sectional study. A total of 10 paired primary NSCLC tissues and adjacent noncancerous tissues were collected from Tianjin Medical University General Hospital for RT-qPCR. Clinical data were extracted for RNA sequencing expression data of the PLAUR gene. The authors performed PLAUR’s prognostic value analysis in NSCLC using The Cancer Genome Atlas (TCGA). The authors then identified ncRNAs contributing to PLAUR overexpression by combining analyses of expression, correlation, and survival. Kaplan-Meier curves and log-rank tests were used to evaluate Overall Survival (OS) and Disease-Free Survival (DFS) differences based on PLAUR expression levels. Finally, the relationship between PLAUR and immune cell infiltration, as well as immune cell biomarkers, was analyzed by immune cell infiltration analysis.

**Results:**

Both the quantitative real-time polymerase chain reaction and the TCGA database results showed significant differences in the expression of PLAUR in NSCLC types. The results showed that patients with a high expression of PLAUR had poorer prognoses, and PLAUR can serve as a prognostic biomarker for patients with NSCLC. The results of studying the regulatory microRNAs (miRNAs) upstream of PLAUR indicated that hsa-miR-340-5p may be the most important regulatory miRNA of PLAUR in NSCLC. Further predictions for upstream long ncRNAs (lncRNAs) in hsa-miR-340-5p indicated that AC008555.6, AC026356.1, TRHDE-AS1, and SNHG14 were the upstream lncRNAs of the has-miR-340-5p/PLAUR axis with the most potential in NSCLC. The expression of PLAUR was significantly correlated with the expression of all immune cells in NSCLC.

**Conclusion:**

Plasminogen activator urokinase receptor was differently expressed and positively correlated with an unfavorable prognosis in NSCLC. The MIRLET7BHG/hsa-miR-127–3p axis was identified as the most likely upstream ncRNA-related pathway for PLAUR in NSCLC. This study provides insights into the molecular mechanism of action of PLAUR in NSCLC, which may help to develop new drugs to regulate PLAUR expression or its function in tumor development and provide new targets for the treatment of NSCLC.

## Highlights

• Significant differences in the expression of PLAUR in NSCLC tumor types.
• PLAUR could serve as a prognostic biomarker for NSCLC patients.
• MIRLET7BHG/hsa-miR-127–3p axis was the upstream pathway for PLAUR.
• PLAUR-mediated carcinogenesis of NSCLC involves tumor immune escape.

## Introduction

Cancer has become the second leading cause of death in humans and is an important public health concern worldwide.^1^ Although there has been a decline in lung cancer incidence and mortality in recent decades, it remains the second most common cancer globally.^2^ It is estimated that Non-Small Cell Lung Cancers (NSCLCs), comprised mainly of Lung Adenocarcinomas (LUADs) and lung squamous cell carcinomas, account for 80 %–85 % of all lung cancers.^3^ Nowadays, with advances in molecular diagnostics, the management of NSCLC has become increasingly personalized.^4^ On a molecular and biological level, NSCLC has been shown to be a collection of multiple diseases. This means that, in addition to oncogenic mutations, NSCLC is also associated with non-oncogenic mutations, such as loss-of-function tumor suppressor mutations.^5^ Consequently, NSCLC requires effective therapeutic targets and promising prognostic biomarkers.

Plasminogen Activator Urokinase Receptor (PLAUR) is involved in many normal and pathological processes related to the activation of cell surface plasminogen and the degradation of extracellular matrices on a local scale.^6^ The PLAUR gene plays an important role in the progression of cancer and is highly expressed in tumors, where its expression is closely associated with tumor invasion and migration.^7^ It has been observed that PLAUR is overexpressed in many cancers and is often associated with poor prognoses and survival rates.^8^ There is evidence that PLAUR works in metastasis, angiogenesis, and inflammatory cell chemotaxis, as well as in extracellular matrix degradation.^9^ Studies suggest that tissue-expressed PLAUR may be a biomarker for cancer progression. However, due to the requirement for tissue specimen availability, the use of PLAUR is limited.^10^ Additionally, abnormal PLAUR expression in tumors has been shown to increase tumor macrophage infiltration, suggesting that there may be a relationship between PLAUR function and tumor immunity.^11^ As yet, there has not been a comprehensive study regarding the expression, prognosis, and mechanism of PLAUR in NSCLC. Furthermore, an investigation of the association between PLAUR and tumor immune infiltration in NSCLC is still pending.

This study systematically investigates the regulatory relationship between PLAUR and ncRNAs in NSCLC and their mechanism of action in tumor immunity. PLAUR expression was found to be significantly correlated with the level of infiltration of a variety of immune cells and the expression of immune checkpoints. This study lays a foundation for the development of personalized therapeutic strategies and immunotherapeutic targets based on PLAUR and its regulatory networks, providing a basis for the targeted treatment of NSCLC in clinical practice.

## Materials and methods

### Clinical case collection

A total of 10 samples of matched primary NSCLC tissues, and their corresponding adjacent noncancerous tissues, were collected from Tianjin Medical University General Hospital. All samples were immediately frozen and saved in liquid nitrogen. Enrolled patients had not received neoadjuvant chemotherapy or radiotherapy. Patients who underwent emergency surgery were excluded. Normal, corresponding, adjacent lung tumor tissues whose distance from the edge of tumors was > 3 cm were regarded as control tissues. All tissue specimens were used after approval from the Ethics Committee of Tianjin Medical University General Hospital (Ethical n° IRB2021-WZ-055). This study followed the STARD guidelines. All patient information was anonymized by assigning unique codes to each sample and data entry, ensuring that no personal identifiers could be traced back to the patients. All researchers involved in the study underwent ethical training and signed confidentiality agreements to prevent the disclosure of any patient information during the study process. The study was conducted under the supervision of the Ethics Committee of Tianjin Medical University General Hospital to ensure adherence to ethical.

### Quantitative real-time polymerase chain reaction

The total RNA in tissues was isolated using RNAzol® RT according to the manufacturer’s instructions (GeneCopoeia, Rockville, MD, USA), and RNA was reverse transcribed into complementary DNA using a reverse transcription kit (Thermo, Waltham, MA, USA). A quantitative real-time polymerase chain reaction (RT-qPCR) was performed according to the instructions of the SYBR® Green kit (TaKaRa, Tokyo, Japan), with Roche LightCycler480® Probes Master reagent (Roche, Basel, Switzerland). Glyceraldehyde 3-phosphate dehydrogenase served as the internal reference control. Relative gene expression levels were calculated using the 2^−ΔΔCt^ method. All primers used were synthesized by Sangon Biotech (Shanghai, China), and the sequences extracted are listed in Table 1.

**Table 1:** Primer sequences for quantitative real-time PCR.

Gene	Primer Sequence (5′→3′)	
GAPDH	Forward	GGAAGCTTGTCATCAATGGAAATC
	Reverse	TGATGACCCTTTTGGCTCCC
PLAUR	Forward	GGGGATTGCCGTGTGGAAGAGT
	Reverse	CTTCAAGCCAGTCCGATAGCTCAGG

### Data acquisition from The Cancer Genome Atlas and profile analysis of plasminogen activator urokinase receptor expression in non-small cell lung cancer

**Fig. 1:**
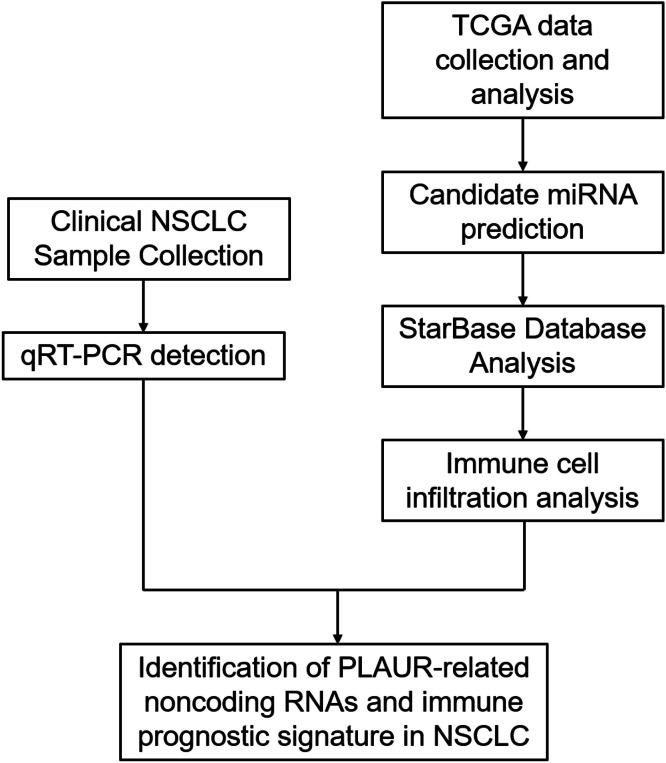
Flow chart.

Plasminogen activator urokinase receptor RNA-sequencing expression profiles and corresponding clinical information for NSCLC were downloaded from The Cancer Genome Atlas (TCGA) dataset (https://genome-cancer.ucsc.edu/).^12^ The log-rank test^13^ was used to compare differences in survival. A timeROC (v. 0.4) analysis was used to compare the predictive accuracy of PLAUR mRNA. For Kaplan-Meier curves,^14^ p-values and a hazard ratio with a 95 % Confidence Interval were generated via log-rank tests and a univariate Cox proportional hazards regression. Statistical analyses were performed using *R* software v. 4.0.3 (*R* Foundation for Statistical Computing, Vienna, Austria).^15^ Statistical significance was established at *p* < 0.05. Experimental procedures are presented in Fig. 1.

### Candidate microRNA prediction

Upstream microRNAs (miRNAs) of PLAUR were predicted using target gene prediction programs. For subsequent analyses, only predicted miRNAs that emerged frequently in one or more programs were considered. Some of these predicted that miRNAs can be considered candidate genes for PLAUR miRNAs.

### StarBase database analysis

With the assistance of the starBase database (http://starbase.sysu.edu.cn/),^16^ miRNA–mRNA and long ncRNA (lncRNA)-miRNA interactions were predicted. With the PLAUR miRNA candidates predicted, the authors used starBase to predict potential lncRNA binding sites for these candidates. In addition, the authors examined miRNAs and lncRNAs in the NSCLC tissue samples and compared them with the control samples. Furthermore, Kaplan-Meier survival analyses were used to assess the prognosis of potential targets. Correlation analyses of miRNA-mRNA, lncRNA-miRNA, and lncRNA-mRNA expression were also conducted in the NSCLC samples.

### Immune cell infiltration of plasminogen activator urokinase receptor in patients with non-small cell lung cancer

To assess the reliability of immune score evaluation results, the authors used an R software package that integrates the Tumor Immune Estimation Resource (https://cistrome.shinyapps.io/timer/).^17^ The R software ‘ggstatsplot’ package was used to draw correlations between gene expression and immune score, and the *R* software heatmap package was used to draw multi-gene correlations. Using the Spearman test,^18^ the authors analyzed the correlation between infiltrating immune cells. An analysis was conducted to examine the relationship between PLAUR expression and levels of immune cell infiltration.

### Plasminogen activator urokinase receptor-related immunomodulators

Transcripts of related genes were selected, and the expression values of these genes were extracted. All of the above analysis methods and *R* packages were implemented with the *R* Foundation for Statistical Computing (2020) v. 4.0.3 software using the ‘ggplot2’ *R* package.

### Statistical analysis

This study used database-derived tools or the *R* language to conduct all statistical analyses. A p-value of < 0.05 and a log-rank of *p* < 0.05 were regarded as statistically significant.

## Results

### Analysis of plasminogen activator urokinase receptor expression in non-small cell lung cancer

**Fig. 2:**
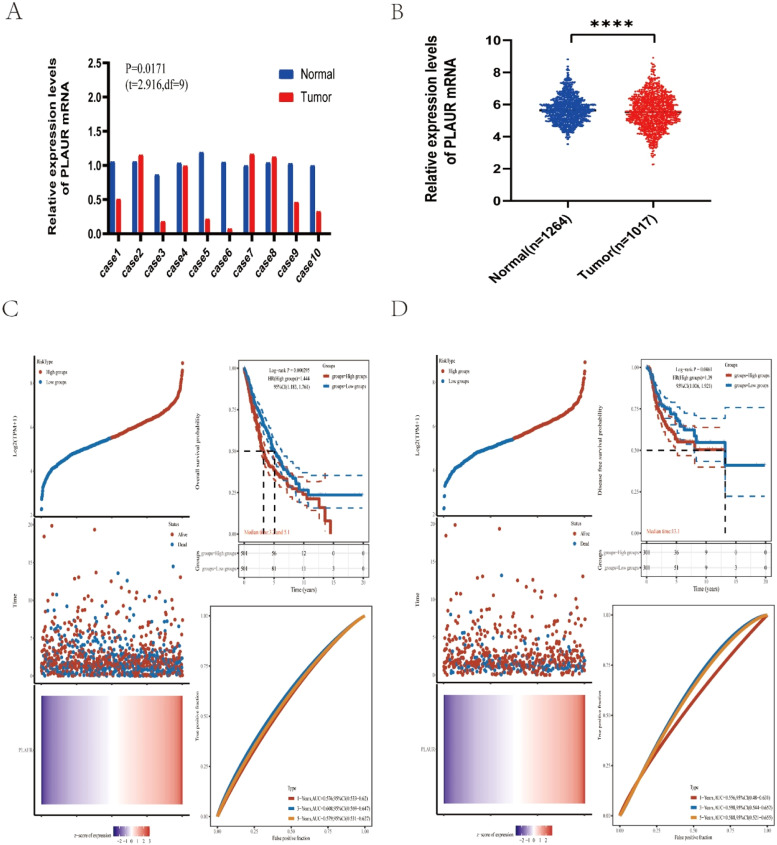
Expression analysis and prognostic values for PLAUR in NSCLC cancers. (A) The expression of PLAUR in 10 paired NSCLC tissues and adjacent noncancerous tissues, as determined by RT-qPCR. (B) PLAUR expression levels in TCGA NSCLC cases, comparing tumor tissues with normal tissues. (C) Kaplan-Meier Overall Survival (OS) plot for PLAUR in NSCLC, with high and low expression groups based on the median value. (D) Kaplan-Meier disease-Free Survival (DFS) plot for PLAUR in NSCLC, with high and low expression groups based on the median value.

To identify the possible roles of PLAUR in NSCLC, the authors first analyzed its expression in 10 NSCLC cases from Tianjin Medical University General Hospital. The results of an RT-qPCR indicated that there was a significant difference in the expression of PLAUR in NSCLC tissues compared with adjacent tissues (Fig. 2A). Using the TCGA dataset, the authors collected 1017 NSCLS tissue samples and 1264 normal tissue samples. Compared with normal samples, there was a significant difference in PLAUR expression in NSCLC tissues (Fig. 2B).

### Prognostic value of plasminogen activator urokinase receptor expression in non-small cell lung cancer

To identify the association between PLAUR and NSCLC tumor prognosis, Kaplan-Meier curves were constructed to evaluate the Overall Survival (OS) and Disease-Free Survival (DFS) of NSCLC cancer types. Based on the median expression value of PLAUR, NSCLC samples were categorized into high- and low-expression groups. Both OS and DFS curves showed that cases with higher PLAUR expression were associated with poor prognoses (Fig. 2C and D). When taken together, PLAUR may be used as a prognostic biomarker for patients with NSCLC.

### Prediction and analysis of plasminogen activator urokinase receptor miRNAs

**Fig. 3:**
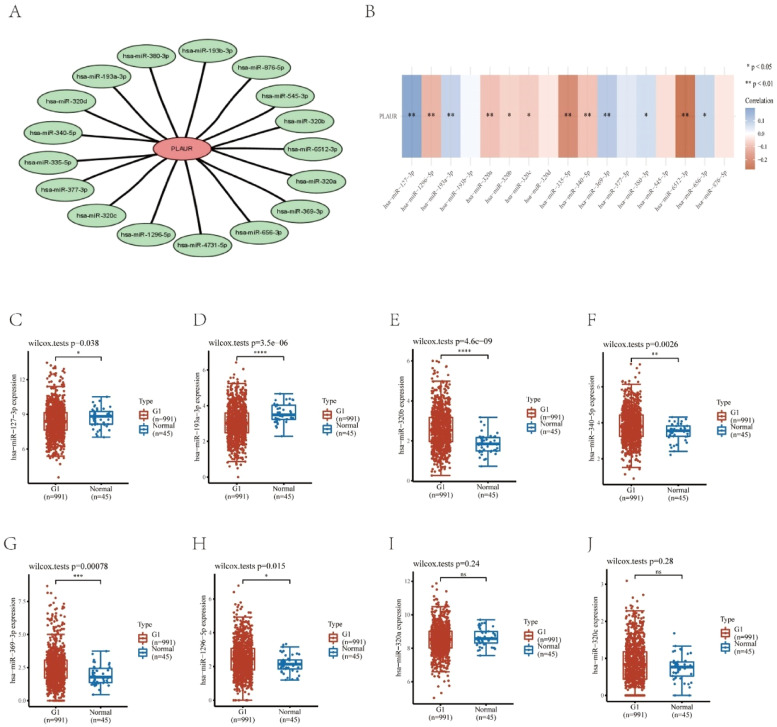
Identification of potential upstream miRNAs of PLAUR in NSCLC. (A) The miRNA-PLAUR regulatory network established by Cytoscape software, showing the interactions between predicted miRNAs and PLAUR. (B) The expression correlation between predicted miRNAs and PLAUR in NSCLC, was analyzed by the starBase database. (C‒J) The expressions of miRNAs determined by the starBase database in NSCLC and control normal samples, highlighting significant differences.

**Fig. 4:**
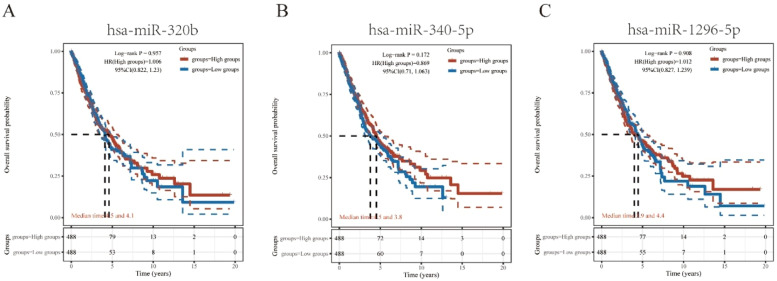
Identification of hsa-miR-340-5p as a potential upstream miRNA of PLAUR in NSCLC. (A‒C) The prognostic value of hsa-miR-320b, hsa-miR-340-5p, and hsa-miR-1296–5p in NSCLC, assessed by Kaplan-Meier curves and log-rank tests.

It has been widely established that ncRNAs play an important role in regulating gene expression. As a first step in determining whether PLAUR is modulated by some ncRNAs, the authors investigated the regulator miRNAs upstream of PLAUR and identified 17 miRNAs. To improve visualization, Cytoscape software (https://cytoscape.org/ Github) was used to establish a miRNA-PLAUR regulatory network (Fig. 3A). According to miRNA’s mechanism of regulation, miRNA and PLAUR should have a negative correlation. The 17 upstream regulatory miRNAs were further analyzed for expression correlation with PLAUR in patients with NSCLC using the starBase database (Fig. 3B; Table 2). As shown, PLAUR was significantly positively correlated with five miRNAs and negatively correlated with seven miRNAs in patients with NSCLC. To further confirm whether these miRNAs influenced NSCLC, their expression levels in NSCLC tissues and in normal tissues were analyzed (Figs. 3C–J), and it was found that a total of six miRNAs, including hsa-miR-127–3p, hsa-miR-193a-3p, hsa-miR-320b, hsa-miR-340-5p, hsa-miR-369–3p and hsa-miR-1296–5p, had significantly different expressions in NSCLC tissues. Among these six significant miRNAs, hsa-miR-127–3p, hsa-miR-193a-3p, and hsa-miR-369–3p were negatively correlated with PLAUR in NSCLC samples compared with normal lung tissue samples. The prognostic value of these three miRNAs in NSCLC was then determined (Fig. 4). Although there was no significant difference in the prognostic value of these three miRNAs, their upregulations were positively linked to patient prognosis, and among them, hsa-miR-340-5p had the greatest prognostic value. Based on these findings, hsa-miR-340-5p is potentially the most important regulatory miRNA of PLAUR in NSCLC.

**Table 2:** The expression correlation analysis between predicted miRNAs and PLAUR.

Gene	miRNA	cor	p-value
PLAUR	hsa-miR–127–3p	0.199118594	2.89E-10^a^
PLAUR	hsa-miR-193a-3p	0.087852543	0.00579676^a^
PLAUR	hsa-miR-320a	−0.094671614	0.002938009^a^
PLAUR	hsa-miR–369–3p	0.106267139	0.000836605^a^
PLAUR	hsa-miR–377–3p	0.044841153	0.15965081
PLAUR	hsa-miR–380–3p	0.06429464	0.043654419^a^
PLAUR	hsa-miR–335–5p	−0.211182475	2.15E-11^a^
PLAUR	hsa-miR-193b-3p	0.014575124	0.647757689
PLAUR	hsa-miR–545–3p	−0.048928706	0.124886408
PLAUR	hsa-miR–656–3p	0.08142503	0.010573046^a^
PLAUR	hsa-miR–340-5p	−0.095462694	0.00270764^a^
PLAUR	hsa-miR–876–5p	−0.035302863	0.26833359
PLAUR	hsa-miR-320b	−0.068346061	0.031968367^a^
PLAUR	hsa-miR-320c	−0.077656959	0.01477578^a^
PLAUR	hsa-miR–1296–5p	−0.120050594	0.000158961^a^
PLAUR	hsa-miR-320d	−0.03596433	0.259462855
PLAUR	hsa-miR–6512–3p	−0.274210476	1.89E-18^a^

a These results are statistically significant.

### Prediction of upstream long non-coding RNAs of hsa-miR-340-5p

**Fig. 5:**
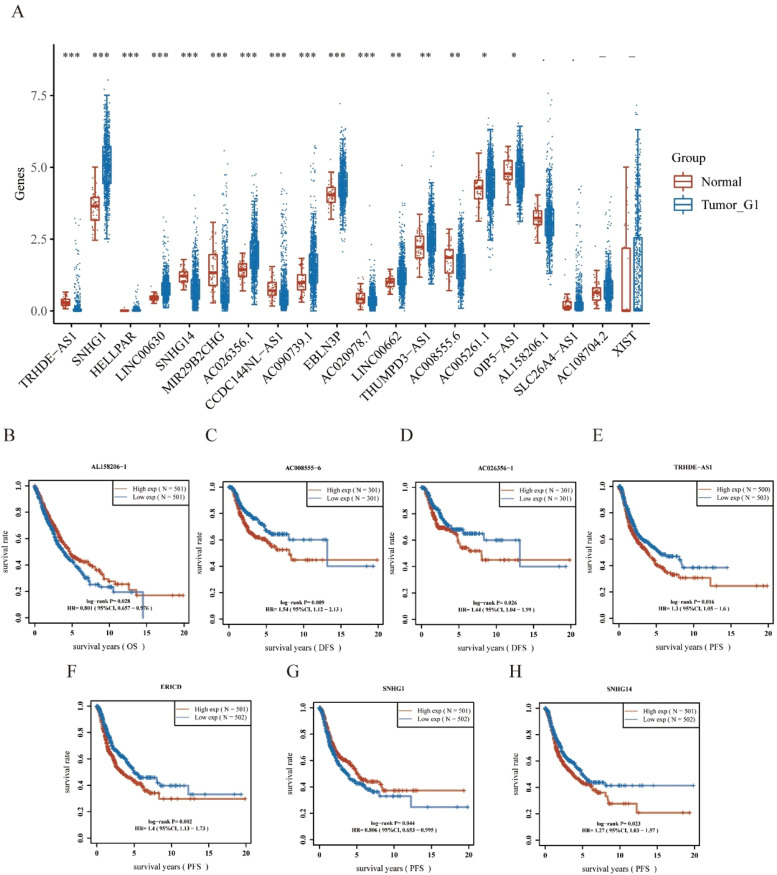
Identification of potential upstream lncRNAs of Hsa-miR-340-5p-PLAUR axis in NSCLC using the StarBase database. (A) The expressions of lncRNAs determined by the starBase database in NSCLC, show differential expression levels. (B‒H) The significant prognostic value of lncRNAs in NSCLC, was assessed by Kaplan-Meier curves and log-rank tests.

Using the starBase database, the authors predicted the upstream lncRNAs of hsa-miR-340-5p and determined 29 possible lncRNAs. The differential expression levels of these lncRNAs in NSCLC were detected (Fig. 5A). Among them, NSCLC samples exhibited a remarkably different regulation of 16 lncRNAs compared with controls. In the subsequent study, 29 lncRNAs were evaluated for prognostic value in NSCLC. As shown in Fig. 5B–H, a total of seven lncRNAs showed different prognostic values in patients with NSCLC. There should be a positive correlation between lncRNA expression and mRNA expression and a negative correlation between miRNA expression and lncRNA expression.^19^ According to differential expression, survival, and correlation analyses, AC008555.6, AC026356.1, TRHDE-AS1, and SNHG14 were predicted to be the upstream lncRNAs of the hsa-miR-340-5p/PLAUR axis with the most potential in NSCLC.

### Correlation of plasminogen activator urokinase receptor with immune cell infiltration in non-small cell lung cancer

**Fig. 6:**
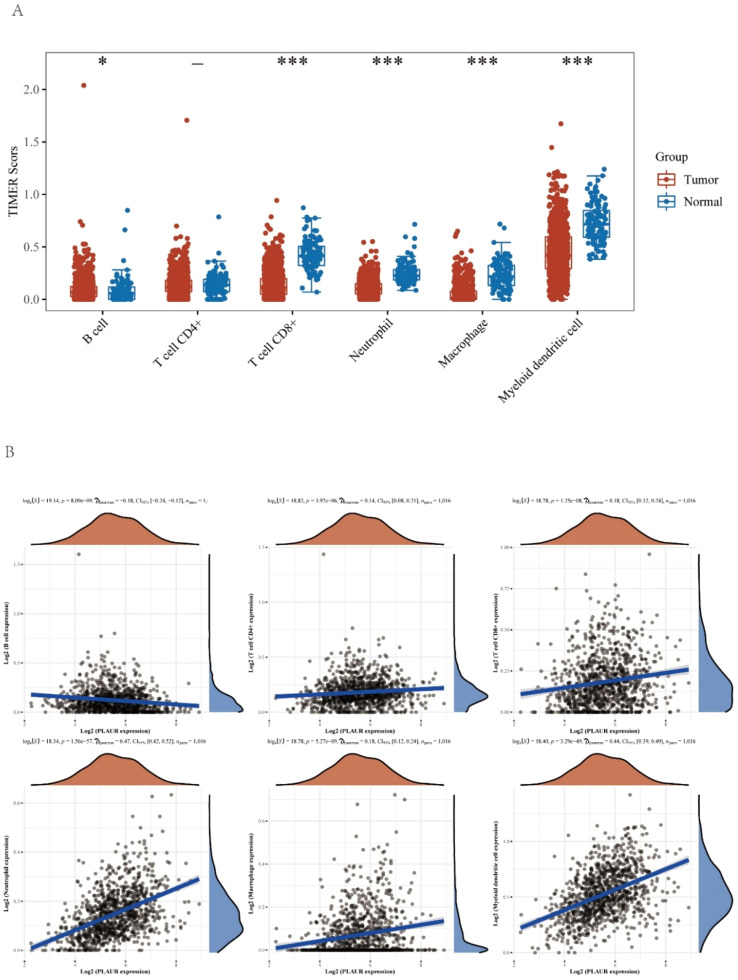
The relationship of immune cell infiltration with PLAUR level in NSCLC. (A) The infiltration level of various immune cells under different copy numbers of PLAUR in NSCLC, visualized by immune cell infiltration analysis. (B) The correlation of PLAUR expression level with B-cell, CD8+ *T*-cell, CD4+ *T*-cell, macrophage, neutrophil, or dendritic cell infiltration level in NSCLC, was analyzed by Spearman correlation tests.

It has been reported that PLAUR plays a critical role as a potential marker in the immune system.^20^ Levels of immune cells infiltration in NSCLC were significantly altered by the copy number of PLAUR (Fig. 6A). The functions and mechanisms of PLAUR can be elucidated by a correlation analysis. Consequently, the correlation between PLAUR expression and the infiltration of different immune cells was examined (Fig. 6B), and a significant positive correlation was found between the expression of PLAUR and the expression of all immune cells in NSCLC, including B-lymphocytes, CD8+ *T*-cells, CD4+ *T*-cells, macrophages, neutrophils, and dendritic cells.

### Correlation of plasminogen activator urokinase receptor expression with immune cell biomarkers in non-small cell lung cancer

**Fig. 7:**
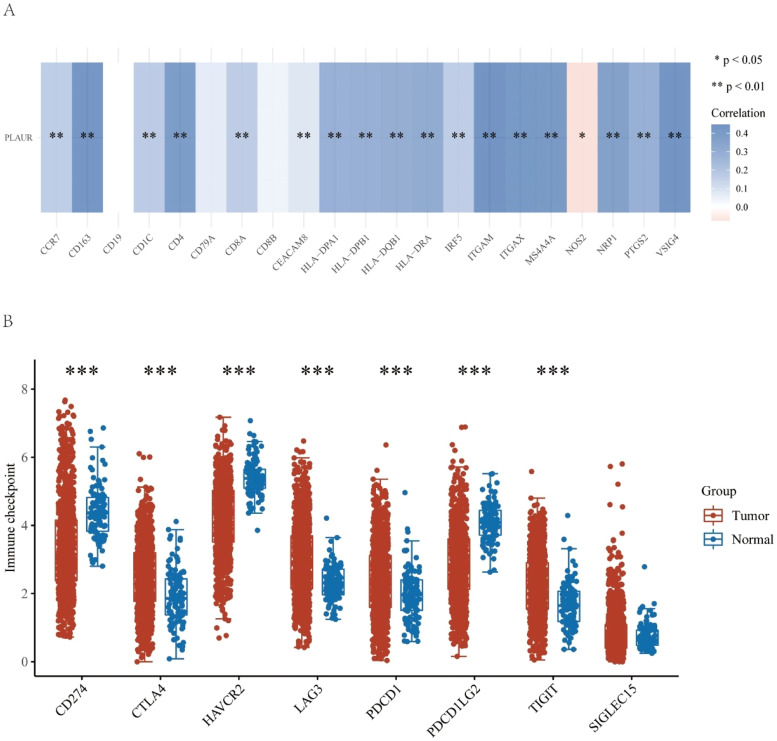
Correlation analysis between PLAUR and immune-related factors in NSCLC. (A) Correlation analysis between PLAUR and biomarkers of immune cells, including B-cells, CD8+ *T*-cells, CD4+ *T*-cells, M1 and M2 macrophages, neutrophils, and dendritic cells. (B) Relationship between PLAUR and immune checkpoints, including CD274, CTLA4, HAVCR2, LAG3, PDCD1, PDCD1LG2, and TIGIT.

This study further explored the role of PLAUR in tumor immunity by determining the correlation of PLAUR expression with immunological markers in NSCLC. As listed in Table 3 and shown in Fig. 7A, PLAUR was significantly positively correlated with CD8+ *T*-cell biomarkers (CD8A), CD4+ *T*-cell biomarkers, M1 macrophage biomarkers (NOS2, IRF5 and PTGS2), M2 macrophage biomarkers (CD163, VSIG4 and MS4A4A), neutrophil biomarkers (CEACAM8, ITGAM and CCR7) and dendritic cell biomarkers (HLA-DPB1, HLA-DPQ1, HLADRA, HLA-DPA1, CD1C, NRP1 and ITGAX) in NSCLC. These results support the hypothesis that PLAUR may be positively related to immune cell infiltration in some cases.

**Table 3:** Correlation analysis between PLAUR and biomarkers of immune cells in NSCLC.

Immune cell	Biomarker	Cor	p-value
B-cell	CD19	0.007562537	0.809648551
	CD79A	0.06129483	0.050683165
CD8^+^T-cell	CD8A	0.162142602	2.00E-07^a^
	CD8B	0.039832451	0.204364103
CD4^+^T-cell	CD4	0.383939568	4.60E-37^a^
M1 macrophage	NOS2	−0.074335135	0.017742647^a^
	IRF5	0.159341361	3.26E-07^a^
	PTGS2	0.287171255	9.25E-21^a^
M2 macrophage	CD163	0.433568097	7.32E-48^a^
	VSIG4	0.43781451	7.14E-49^a^
	MS4A4A	0.408764818	3.08E-42^a^
Neutrophil	CEACAM8	0.081297884	0.009494073^a^
	ITGAM	0.447515036	3.09E-51^a^
	CCR7	0.16089805	2.49E-07^a^
Dendritic cell	HLA-DPB1	0.284738332	2.01E-20^a^
	HLA-DQB1	0.291530544	2.25E-21^a^
	HLA-DRA	0.31054357	3.56E-24^a^
	HLA-DPA1	0.28185114	5.01E-20^a^
	CD1C	0.165781785	1.05E-07^a^
	NRP1	0.369385977	3.13E-34^a^
	ITGAX	0.412051784	5.89E-43^a^

a These results are statistically significant.

### Relationship between plasminogen activator urokinase receptor and immune checkpoints in non-small cell lung cancer

Since immunotherapy has significantly transformed cancer treatment, the authors investigated whether PLAUR is related to immunomodulators. As a preliminary investigation into the relationship between high- and low-PLAUR expression groups in established immunotherapies, the authors examined the association between high and low PLAUR expression in a number of established immune-related signatures, including CD274, CTLA4, HAVCR2, LAG3, PDCD1, PDCD1LG2, TIGIT and SIGLEC15. The authors found that PLAUR expression was significantly positively correlated with CD274, CTLA4, HAVCR2, LAG3, PDCD1, PDCD1LG2 and TIGIT (Fig. 7B). The results of this study suggest that the PLAUR-mediated carcinogenesis of NSCLC involves tumor immune escape.

## Discussion

In this study, the authors first enrolled patients with NSCLC from Tianjin Medical University General Hospital. Their primary NSCLC tissues and corresponding, adjacent noncancerous tissues were collected, and RT-qPCR was performed to detect the relative expression levels of PLAUR genes. The authors found that there was a significant difference in the expression of PLAUR in NSCLC tissues compared with adjacent tissues. The authors then used TCGA data to analyze the expression of PLAUR in NSCLC. Transcriptome sequencing data and the corresponding clinical features of NSCLCs such as lung squamous cell carcinoma and LUAD were downloaded from TCGA, and there was also a significant difference in PLAUR expression. Finally, a survival analysis showed that patients with NSCLC with a high PLAUR expression had poor OS and DFS. A previous study indicated that only patients with colon and lung cancers showed a significant survival disadvantage due to high PLAUR gene expression.^10^ The present results confirm the tumourigenicity of PLAUR in NSCLC in alignment with this previous report.

An overview of lncRNAs, miRNAs and mRNAs in the corpus luteum changing with the breed was compiled, and, following this, miRNA-mRNA and lncRNA-mRNA interactions were predicted. A competing endogenous RNA (ceRNA) network was constructed based on these interactions.^21^ According to the ceRNA hypothesis, lncRNA can interact with miRNA to regulate target gene expression.^22^ The analysis of the starBase database predicted 17 potential upstream regulator miRNAs that could bind to PLAUR. In NSCLC, the majority of these miRNAs may act as tumor suppressors. For example, overexpression of miR-127–3p could rescue the effects of FOXD3-AS1 on NSCLC progression^23^; miR-193a-3p can promote lung cancer cell invasion by activating STAT3-mediated epithelial-mesenchymal transitions through exosomes^24^; and miR‐320b targeting BMI1 regulated by NR2F2‐AS1 may influence the cell proliferation, invasion, and apoptosis of NSCLC.^25^ Following an analysis of the 17 predicted miRNAs, hsa-miR-340-5p was identified as the most probable PLAUR miRNA, was negatively associated with PLAUR expression, and was overexpressed and predicted a bad outcome in NSCLC. Previous studies show that the miR-340-5p/NET1 axis-dependent effect of baicalin can suppress lung cancer progression.^26^ By targeting ZNF503, miRNA-340-5p suppresses the growth and metastasis of NSCLC cells.^27^ A reduction in hsa-miR-340-5p expression might influence cisplatin resistance by mediating SOX2 expression in small-cell lung cancer cells.^28^ As a result of miR-340-5p regulation, PNO1 plays an important role in LUAD progression through Notch signaling.^29^

Subsequently, 29 upstream potential lncRNAs of the miR-340-5p/PLAUR axis were discovered. Upregulated lncRNAs with the most potential, including AC008555.6, AC026356.1, TRHDE-AS1, and SNHG14, were identified through expression, survival, and correlation analyses. The findings of emerging research suggest that these lncRNAs are crucial to the progression of various cancers: lncRNA-AC026356.1 is upregulated by m6A to promote cancer stem cell maintenance in LUADs^30^; AC026356.1 in LUAD acted as a ceRNA of miR-30d-5p and might be the possible regulatory miRNA of CAPZA.^31^ The TRHDE-AS1/PKIA network participated in the prognosis of hepatocellular carcinoma patients.^32^ By regulating the PD-1/PD-L1 checkpoint, there was a positive feedback loop between SNHG14/miR-5590–3p/ZEB1 that led to the progression of diffuse large B-cell lymphomas and immune evasion.^33^ The expression level of lncRNA SNHG14 in endometrial carcinoma tissues is significantly higher, and the presence of this factor has been found to be significantly associated with tumor size, pathological stage and poor prognosis in endometrial carcinoma patients.^34^ In colorectal cancer, SNHG14 modulates the miR-519b-3p/DDX5 axis to promote cell proliferation and invasion.^35^ Based on the above results, the authors considered the possible lncRNA- hsa-miR-340-5p/PLAUR axis as a potential regulatory pathway in NSCLC.

Tumor immune cell infiltration has been linked to the effectiveness of chemotherapy, radiotherapy, or immunotherapy and the prognosis of patients with cancer in numerous studies.^36^ The present work suggests that PLAUR expression significantly correlates with the expression of all immune cells in NSCLC, including B-lymphocytes, CD8+ *T*-cells, CD4+ *T*-cells, macrophages, neutrophils, and dendritic cells. There was also a significant positive correlation between PLAUR expression and a variety of immune cell biomarkers. The results of this study suggest that PLAUR may influence the progression of NSCLC mediated by tumor immune infiltration. It is also crucial that immune checkpoints are sufficiently expressed in the tumor microenvironment for immunotherapy to be effective.^37^ Thus, the authors also investigated the relationship between PLAUR and immune checkpoints. The results demonstrated that PLAUR expression was significantly positively correlated with CD274, CTLA4, HAVCR2, LAG3, PDCD1, PDCD1LG2, and TIGIT, indicating that targeting PLAUR might increase the efficacy of immunotherapy in NSCLC. The positive correlation between PLAUR expression and immune checkpoints such as CD274, CTLA4, and PDCD1 is particularly noteworthy. These immune checkpoints play a pivotal role in immune evasion by tumors, and their upregulation is often associated with poor prognosis in cancer patients. The expression of CD274 on tumor cells and immune cells can inhibit T-cell activation, thereby suppressing antitumor immune responses. Similarly, CTLA4 and PD-1 are key negative regulators of T-cell activation, and their upregulation can lead to immune tolerance and tumor progression.^38^ The present findings suggest that PLAUR may contribute to immune evasion in NSCLC by upregulating these immune checkpoints. Targeting PLAUR could potentially enhance the efficacy of immune Checkpoint Inhibitors (ICIs) in several ways. First, by reducing PLAUR expression, the authors may indirectly lower the expression levels of immune checkpoints, thereby sensitizing tumors to ICIs. Second, the inhibition of PLAUR could disrupt the tumor microenvironment, leading to increased immune cell infiltration and enhanced antitumor immunity. This dual effect could potentially improve the response rates and overall outcomes of patients receiving ICIs. Moreover, the correlation between PLAUR and immune checkpoints provides a rationale for combination therapies targeting both PLAUR and immune checkpoints. For instance, combining PLAUR inhibitors with anti-PD-1 or anti-CTLA4 antibodies may achieve a synergistic effect, enhancing the antitumor immune response and overcoming resistance to single-agent ICIs. Future studies should explore the potential of such combination therapies in preclinical models and clinical trials.

The main strengths of this study are that, for the first time, the authors identified the AC008555.6, AC026356.1, TRHDE-AS1, or SNHG14-hsa-miR-340-5p/PLAUR axis in NSCLC. The present results indicate that the presence of PLAUR in tumors could contribute to the development of oncogenic functions through the infiltration of tumor immune cells and the expression of immune checkpoints. However, the study also has a few limitations. First, the sample size consisted of only 10 cases, and there may have been some degree of bias when conducting data statistics. Further studies with more sample sizes collected at multiple sites and fully confirming the conclusions of this study are still needed in the future. Second, the proposed SNHG14-hsa-miR-340-5p/PLAUR axis is based only on correlation, and its correctness still needs further verification. Its mechanism and function still need further in-depth experimental research. Future studies should focus on functional assays to demonstrate the direct interactions between these molecules. For instance, knockdown or overexpression experiments of SNHG14, hsa-miR-340-5p, and PLAUR in NSCLC cell lines could elucidate their regulatory roles in tumor progression and immune cell infiltration. Additionally, in vivo studies using animal models would further validate the therapeutic potential of targeting this axis. In addition, clinical validation, and verification of PLAUR’s application in clinical practice need to be conducted.

## Conclusion

The PLAUR gene was differently expressed and positively correlated with an unfavorable prognosis in NSCLC. The authors constructed a PLAUR-related ceRNA regulatory network for NSCLC, namely the AC008555.6-, AC026356.1-, TRHDE-AS1- or SNHG14-hsa-miR-340-5p/PLAUR axis. The current study also suggested that PLAUR could exert its tumourigenic effects by regulating tumor immune cell infiltration and immunomodulatory function. By identifying PLAUR-associated ncRNA regulatory networks, this study provides the basis for the development of personalized therapeutic strategies against specific molecular markers. Understanding the interactions between these molecules could aid in the design of targeted therapies for specific patient populations and in the development of new drugs to modulate PLAUR expression or its function in tumor development.

Clinically, the identified ncRNA-PLAUR axis holds significant promise for therapeutic intervention. Targeting the ncRNAs involved in this axis, such as AC008555.6, AC026356.1, TRHDE-AS1, or SNHG14, could potentially disrupt the regulatory mechanisms that lead to PLAUR overexpression, thereby reducing tumor progression and improving patient outcomes. Additionally, given the strong correlation between PLAUR expression and immune cell infiltration, as well as immune checkpoints, developing PLAUR-targeted immunotherapies could enhance the efficacy of existing cancer treatments. These findings open new avenues for the development of ncRNA-based therapies and PLAUR-targeted approaches, offering hope for more effective and personalized treatment options for patients with NSCLC.

## Ethics approval and consent to participate

All tissue specimens were used after approval from the Ethics Committee of Tianjin Medical University General Hospital (n° IRB2021-WZ-055). The participants provided their written informed consent to participate in this study.

## Authors’ contributions

Conception and design of the work: Cheng Y, Bao SH, Zhang H; Methodology: Cheng Y, Bao SH, Zhang H, Zhang YF; Data collection: Li XJ, Li HY, Jia XW, Sun QQ; Supervision: Song ZQ; Analysis and interpretation of the data: Zhang YF, Li XJ, Li HY, Jia XW, Sun QQ; Statistical analysis: Cheng Y, Bao SH, Zhang H; Drafting the manuscript: Cheng Y, Bao SH, Zhang H, Song ZQ; Critical revision of the manuscript: all authors; Approval of the final manuscript: all authors.

## Funding

The present study was funded by the Project of Tianjin National Natural Science Foundation 24JCZDJC01030,the Haihe Laboratory of Cell Ecosystem Innovation Fund HH24KYZX0010,the project of Beijing Association of Holistic Integrative Medicine Clinical Research Funding Program Fund ZHKY-2025-1869（C003) and Health Commission municipal level selected project science fund of Hebei Provincial .

## Declaration of competing interest

The authors declare that the research was conducted in the absence of any commercial or financial relationships that could be construed as a potential conflict of interest.

## Data Availability

The PLAUR RNA-sequencing expression profiles and corresponding clinical information for NSCLC were downloaded from the TCGA dataset ().https://genome-cancer.ucsc.edu/
